# Patient and Public Perceptions of Artificial Intelligence in Breast Imaging and Clinical Decision-Making: An Exploratory Cross-Sectional Survey Study †

**DOI:** 10.3390/diagnostics16091376

**Published:** 2026-05-01

**Authors:** Alia Hussein, Mariam Rizk, Kefah Mokbel, Amtul R. Carmichael

**Affiliations:** 1 The London Breast Institute, Princess Grace Hospital, London W1G 9QP, UK; aliaomerhussein@gmail.com (A.H.); kefah.mokbel@hcahealthcare.co.uk (K.M.); 2 University Hospital of Derby and Burton Queen’s Hospital, Burton on Trent DE13 0RB, UK

**Keywords:** artificial intelligence, breast imaging, clinical decision-making, triage

## Abstract

**Background/Objectives**: Artificial intelligence (AI) shows promise in supporting mammography interpretation and triaging referrals, potentially enhancing breast screening. However, successful AI integration depends on patient acceptance and trust. This study explores patient and public perceptions of AI in breast imaging and clinical decision-making to identify knowledge gaps and guide communication strategies. **Methods**: Paper surveys were distributed to women attending the Breast Care Unit at Queen’s Hospital, Burton, and the London Breast Institute between August and December 2025. Demographic data, levels of trust and comfort with AI, and concerns about AI were collected. Responses were analysed using descriptive statistics, Pearson’s Chi-square tests with Cramér’s V and thematic analysis. **Results**: One hundred and twenty participants completed the survey. Fifty percent would accept AI alongside clinicians for interpretation of mammograms or ultrasound scans, significantly associated with no previous breast cancer diagnosis (*p* = 0.02; Cramér’s V = 0.22, 2 degrees of freedom (df)) and technological comfort (*p* < 0.001; Cramér’s V = 0.42, 1 df). Lower acceptance was found among those with prior diagnosis and low comfort with technology. Acceptance of AI-assisted triage (44.5%) was also significantly associated with technological comfort (*p* = 0.008; Cramér’s V = 0.30, 1 df). Eighty percent reported no knowledge of AI use in breast clinics, and only 37% would trust AI findings. Qualitative analysis identified three themes: (1) clinician oversight as indispensable, (2) the knowledge gap as a barrier to acceptance, and (3) concerns about operational risks and accountability. **Conclusions**: Although patients were generally receptive to AI, acceptance was conditional on clinician supervision. Limited awareness and concerns about diagnostic accuracy remain barriers to implementation. Educational initiatives should precede widespread adoption to support informed and confident patient acceptance of AI-assisted imaging and decision-making.

## 1. Introduction

In 2022, 2.3 million women were diagnosed with breast cancer worldwide, with incidence steadily increasing since the mid-2000s [1,2]. Breast cancer is the most common cancer in women in the United Kingdom (UK) [3]. The triennial screening programme for women aged 50–71 aims to support early detection and reduce mortality [4,5]. However, screening may contribute to overdiagnosis, overtreatment, and increased anxiety [6,7].

Double-reading of digital mammograms remains subject to interpretive variability, occasionally leading to missed diagnoses, false positives and recalls for additional imaging [8]. These impose patient distress, healthcare burden and opportunity costs [9,10]. Interval breast cancers (IBCs), detected between routine mammograms, represent an ongoing challenge to screening programme effectiveness [11].

Artificial intelligence (AI) refers to computational algorithms that mimic human intelligence in tasks such as learning, pattern recognition and decision-making. AI applications can support mammography reading, malignancy risk prediction, and referral triage [12,13]. The UK shortage in the radiology workforce has accelerated interest in mitigation strategies including non-radiologist readers and AI tools for mammography interpretation [14,15,16]. Recent research found that AI-detected breast cancers seemed larger and more invasive by the time of detection by human readers, implying potential to recognise IBCs at an earlier stage. The study also found that mammography interpretation by one human reader and AI resulted in increased breast cancer detection in comparison to double radiologist reading [17].

Despite AI’s promising applications, successful integration depends critically on patient acceptance, trust, and understanding [18]. International research suggests patients generally prefer AI as an adjunct rather than a replacement for clinicians [19,20,21].

At present, limited UK-specific research exists on patient perceptions of AI in breast care and clinical decision-making. This study therefore assesses patient and public comfort, concerns, and trust regarding the use of AI in breast imaging and clinical decision-making. We hypothesise that older age, previous cancer diagnosis, and lower comfort with technology will be associated with lower acceptance of AI in breast care.

## 2. Materials and Methods

A 14-question paper questionnaire written in English was distributed to attendees of two breast clinics in the UK, the Breast Care Unit at Queen’s Hospital, Burton, and the London Breast Institute, Princess Grace Hospital, between August and December 2025.

**Figure 1:**
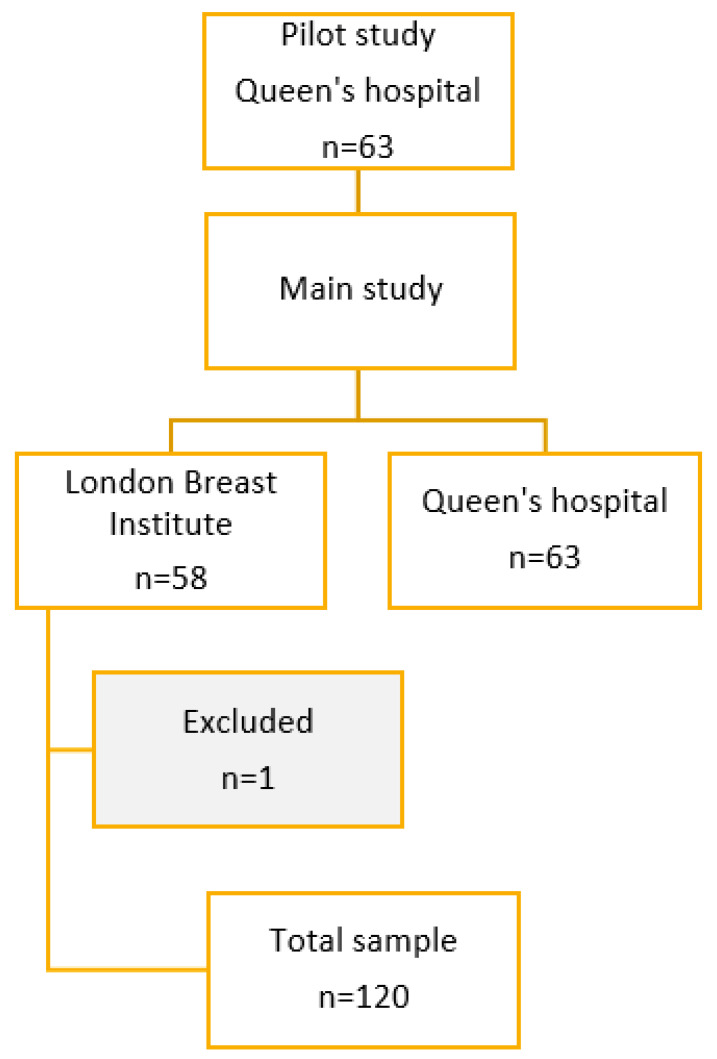
Study flowchart of survey responses from breast clinics at Queen’s hospital, Burton, and London Breast Institute. Out of 121 respondents, 1 response was excluded due to age exclusion criteria.

The questionnaire was initially piloted in 63 respondents from Queen’s Hospital, Burton, which were included in the final dataset post-minor revisions (Figure 1). The optionality of the following demographics questions of age, gender and previous cancer diagnosis were removed.

A formal power calculation was not undertaken. Sample size (*n* = 120) was determined by pragmatic considerations of clinic attendance during the study period. Multiple-testing correction was not conducted due to the exploratory design of the study.

The hypothesis-generating survey assessed perceptions of AI use in breast care including knowledge of AI, comfort with AI-assisted imaging interpretation, comfort with AI-assisted triage, trust in AI findings, and concerns about AI in clinical care. Demographic data included age, gender, previous cancer diagnosis and familiarity with technology. Majority of questions were single-selection multiple choice questions with few multiple selection questions and few open-response questions. There was no time limit to complete the survey, with an anticipated completion time of 5 min. Patients under 18 years of age and over 85 years of age were excluded. Incomplete questions were excluded, with incomplete being defined as questions for which no response was given, treated as missing data. To address missing data, we used pairwise deletion, in which all available data was used in analyses and missing information was eliminated. This resulted in differing sample sizes across statistical evaluations. This method was selected to minimise the loss of information and to preserve statistical power.

Data were analysed using descriptive statistics, inferential statistics and Pearson’s Chi-square tests with Cramér’s V coefficient. Statistical significance was indicated by a *p*-value less than 0.05. Strength of associations were indicated by effect sizes as follows: 0.1 (small); 0.3 (medium), 0.5 (large) at 1 degree of freedom (df) and 0.07 (small); 0.21 (medium), 0.35 (large) at 2 df [22,23]. Likert scale responses were condensed to meet Chi-square assumptions (neutral and “don’t know” responses excluded from acceptance vs. non-acceptance analyses), which may have reduced statistical sensitivity [24]. Responses including very/fairly comfortable with AI use indicated acceptance of AI. Free-text responses were analysed using thematic analysis. Responses were independently coded by two reviewers (authors one and two), and themes were derived iteratively and reflexively through consensus discussion.

This study was reviewed and approved by the institutional board of the London Breast Institute. As the prospective questionnaire was conducted as part of an institutional service evaluation, formal approval from an external Research Ethics Committee was not required, in accordance with UK NHS Health Research Authority (HRA) guidance [25]. Participation was anonymous and voluntary, and completion of the questionnaire was considered informed consent.

## 3. Results

### 3.1. Demographics

A total of 120 participants completed the survey, excluding one survey omitted due to age exclusion criteria. A total of 115 participants provided gender demographic data, with 103 female (89.6%) and 12 male (10.4%) (Table S1). A total of 73.3% of the participants were patients (*n* = 120). Most respondents were between 25 and 74 years of age (90.7%). Eighty-two percent of participants felt comfortable using technology (Table S1).

Before participating in the survey, most respondents had heard about AI being used in healthcare settings (Table S2). The majority of participants reported no knowledge of how AI may be used in breast clinics and minimal trust in AI findings. These results align with prior work demonstrating limited awareness as a barrier to adoption.

### 3.2. Perceptions About AI in Breast Imaging and Clinical Decision-Making

Among the survey population, half accepted AI-assisted mammogram or ultrasound interpretation alongside clinicians. This is consistent with international evidence demonstrating preference for combined AI–radiologist reading (Table S3). Close to half (44.5%), accepted AI-assisted triage.

Among those that accepted AI-assisted screening (*n* = 58), 96.6% were comfortable with technology, whereas 2% were uncomfortable with technology. Acceptance (*n* = 58) was 74.1% in the 46 and over age group; meanwhile, 25.9% accepted in the below-46 age group. Acceptance (*n* = 59) was greater in participants without a previous breast cancer diagnosis (61%) than those with a diagnosis (25.4%).

Among those that accepted AI-assisted triage (*n* = 52), 92.3% were comfortable with technology, whereas 7.7% were uncomfortable with technology. Acceptance was 73.1% in the 46 and over age group; meanwhile, 26.9% accepted AI use in the below-46 age group. Acceptance was greater in participants without a previous breast cancer diagnosis (59.6%) than those with a diagnosis (30.8%).

Sixty-nine percent of participants would accept AI use in their future breast care (Table S3). Among those that accepted (*n* = 81), 60.5% were between ages 46 and 74, 56.8% had no previous diagnosis, and 86.4% were comfortable with technology.

Concerns were common, including fear of diagnostic mistakes (73.5%), AI replacing doctors (63.3%), and privacy concerns (39.3%) (Table S4). This echoes qualitative findings from UK screening-eligible populations. Most participants (78%) reported that clinician confirmation of AI findings would increase comfort, reinforcing that acceptance is conditional on oversight. Participants also reported increased comfort knowing AI had been tested in large trials (42.4%) and if they had more information about how AI works (39.8%).

### 3.3. Statistical Analysis

Acceptance of AI screening was significantly associated with no previous breast cancer diagnosis (*p* = 0.02) and technological comfort (*p* < 0.001) but was not associated with age (*p* = 0.36) (Table 1). Comparably, acceptance of AI-assisted triage was significantly associated with technological comfort (*p* = 0.008), yet independent of clinical history (*p* = 0.32) and age (*p* = 0.32) (Table 2).

### 3.4. Main Qualitative Themes

**Figure 2:**
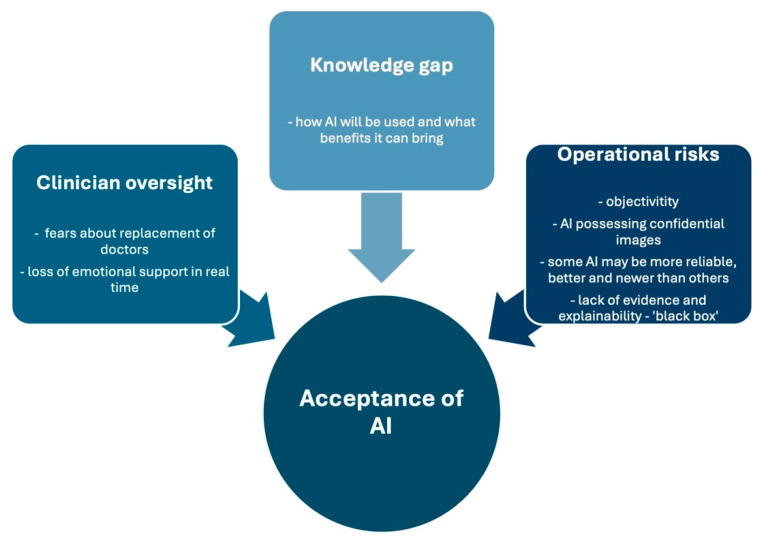
Participant concerns and key patterns surrounding AI that may influence the acceptance of AI, based on qualitative analysis. Concerns include loss of doctor–patient relationship and emotional care, the role of AI in breast clinics and operational risks of AI.

Three qualitative themes emerged: clinician oversight as indispensable, the knowledge gap driving uncertainty, and concerns about operational risks and accountability (Figure 2). The following section presents the key qualitative findings including patient quotes.

*Theme 1: Clinician oversight as indispensable*

There was significant consensus regarding the importance of doctor involvement in AI programmes, including screening and diagnosis. Participants consistently viewed AI as “a tool to optimise, rather than replace doctor assessment”. There was a perception that AI has a “lack of empathy, discernment and other human attributes.” This underscores the importance of the emotional care provided by clinician engagement. It also highlighted that attendance at breast clinics is proof that “human interaction is imperative to mental wellbeing.” The general sentiment was that human connection, alongside the ability to ask follow-up questions, cannot be provided by AI algorithms.

*Theme 2: The knowledge gap driving uncertainty*

Our finding that 80% of participants lacked knowledge about AI use in breast clinics, combined with frequent responses of “don’t know”, suggests a critical knowledge gap. The knowledge disparity surrounding AI in breast imaging acts as a barrier to AI acceptance. How AI will be used, what roles it will fill and what benefits it may bring to the current system were noted. Participants expressed a lack of understanding as a cause for uncertainty and ambivalence. One respondent stated, “if I had more information and understood AI, I would have a different outlook, but I don’t know anything about it”. This indicates that education must precede implementation.

*Theme 3: Concerns about operational risks and accountability*

Concerns about the accuracy, reliability and accountability of AI were apparent, with participants communicating that AI can make “mistakes” and “errors” and that “all AI is not equal”. This is compounded by the perceived novelty of medical AI programs. There was a general sentiment that research into AI in breast imaging should be undertaken and disseminated. In addition, “cyber-attacks”, “confidentiality”, “objectivity”, “data security” and “data privacy” were among the concerns over operational risks of AI.

## 4. Discussion

This study provides UK-based evidence on patient and public perceptions of AI integration into breast imaging and clinical decision-making. Overall, receptivity towards AI was substantial, with most participants willing to accept AI in future breast care, half accepting AI-assisted breast imaging and 44.5% accepting AI-assisted triage. However, acceptance was conditional, with strong emphasis on clinician oversight. The strongest predictor of AI acceptance was technological comfort, consistent with research on the “AI divide” and the role of digital literacy in healthcare technology adoption [26,27]. The significant association between technological comfort and AI acceptance (*p* < 0.001) suggests that general digital literacy programmes may indirectly improve receptivity to AI in healthcare, representing a potentially modifiable factor for increasing acceptance [28].

Our findings align with international surveys reporting preference for AI as a second reader rather than an autonomous decision-maker, as seen in a 2026 study with 61% of patients opting for mixed AI–radiologist readings [29]. Studies reported that AI adoption in breast imaging was more readily accepted than AI-assisted triage, which aligns with our findings [30].

UK qualitative research similarly highlights concerns about errors, the importance of human reassurance and the requirement for further clinical trials [31]. UK studies also found that women of screening age and above were more likely to have positive views about AI-assisted mammogram reading than younger women, as observed in our study with the majority between ages 46 and 74 [32].

Age was not significantly associated with AI acceptance and thus was not a predictor in our sample. This may indicate that comfort with technology is more influential than chronological age or reflect sample homogeneity. Further research is required to ascertain whether digital literacy may predict acceptance of AI.

Lack of previous breast cancer diagnosis was significantly associated with AI acceptance yet minimally reported within the existing literature. Some studies had excluded women with current or previous breast cancer diagnoses altogether [30]. This may imply that previous cancer diagnoses may increase apprehension surrounding AI in breast imaging. Future research should assess the relationship between previous cancer diagnosis and perceptions further.

Concerns about diagnostic errors and accountability relate to the black-box phenomenon, where AI mechanisms are not easily interpretable [33]. Explainable AI frameworks may improve trust and promote acceptance by increasing transparency and validation [34,35,36].

Overall, our research illustrates AI as a highly functional tool with the potential to significantly enhance breast screening. This study’s findings indicate a strong demand for educational initiatives that should focus on (1) explaining AI’s role as a supportive tool rather than a replacement for clinicians; (2) providing transparent information about validation studies and accuracy rates; and (3) addressing specific concerns about diagnostic errors through clear communication about oversight mechanisms. Recommendations for breast screening programmes include the introduction of these interventions, tailored separately for (1) patients and (2) clinicians, within NHS settings. Implementation strategies include targeted education for healthcare professionals, pre-screening patient–doctor discussions, and digital infographics or decision aids using the NHS application and website.

This study adds to the research about how AI can be introduced for women of different ages and with prior cancer diagnoses, as mentioned in the UK National Screening Committee (NSC) external review [37]. Following evidence from the review, additional studies on the perceptions of medical students and clinicians are required to inform educational strategies and increase acceptance and future compliance with AI technologies in breast screening.

Limitations include modest sample size, selection bias (clinic attendees may differ from the general screening population), and reduced statistical power from Likert condensation [38]. The absence of formal questionnaire validation may have reduced the reproducibility of this study and the robustness of the research outcomes. Pairwise deletion also presents limitations including inconsistent sample sizes, reducing external validity. Additionally, we did not adjust for multiple comparisons which may have increased the probability of Type I errors. Nearly 82% of participants were comfortable with technology, possibly biasing results toward greater acceptance of AI-assisted diagnosis and triage. The findings may not apply to those with lower digital literacy. Future studies should use surveys in different languages and incorporate broader demographics, including race and education level, to avoid reinforcing inequities [39,40]. Finally, with AI being a rapidly evolving field, the findings reflect the current context and time in which this study was conducted and offer a preliminary insight into the subject matter.

## 5. Conclusions

We found that while there is meaningful receptivity to AI in breast care, acceptance is not uniform and is moderately influenced by prior clinical experience and familiarity with technology. Limited knowledge remains a key barrier to adoption. Addressing patient and public concerns through transparent communication and patient-centred implementation strategies is critical for effective integration of AI into breast clinics.

## Figures and Tables

**Table 1:** Determinants of AI acceptance of AI-assisted screening including technological comfort, prior cancer and age. *p*-values derived from Pearson’s Chi-square test, with Cramér’s V coefficient used to indicate strength of associations. The effect sizes used are as follows: 0.1 (small); 0.3 (medium), 0.5 (large) at 1 degree of freedom (df) and 0.07 (small); 0.21 (medium), 0.35 (large) at 2 df [22,23]. *n* varies due to pairwise deletion.

	Acceptance of AI-Assisted Screening	Non-Acceptance	*p*-Value	Cramér’s V	Degrees of Freedom
**Comfort with technology**	*n* = 58	*n* = 20	<0.001	0.42	1
Yes	56	11			
No	2	9			
**History of breast cancer**	*n* = 59	*n* = 21	0.02	0.22	2
Prior cancer	15	7			
No prior cancer	36	9			
No prior cancer, but have had concerns	8	5			
**Age**	*n* = 58	*n* = 21	0.36	0.14	2
18–35	5	4			
36–60	34	13			
61–85	19	4			

**Table 2:** Determinants of AI acceptance of AI-assisted triage including technological comfort, prior cancer and age. *p*-values derived from Pearson’s Chi-square test, with Cramér’s V coefficient used to indicate strength of associations. The effect sizes used are as follows: 0.1 (small); 0.3 (medium), 0.5 (large) at 1 degree of freedom (df) and 0.07 (small); 0.21 (medium), 0.35 (large) at 2 df [22,23]. *n* varies due to pairwise deletion.

	Acceptance of AI-Assisted Triage, *n*	Non-Acceptance, *n*	*p*-Value	Cramér’s V	Degrees of Freedom
**Comfort with technology**	*n*= 52	*n* = 26	0.008	0.30	1
Yes	48	18			
No	4	8			
**History of breast cancer**	*n* = 52	*n* = 26	0.32	0.14	2
Prior cancer	16	8			
No prior cancer	31	12			
No prior cancer, but have had concerns	5	6			
**Age**	*n* = 52	*n* = 26	0.32	0.14	2
18–35	4	5			
36–60	31	15			
61–85	17	6			

## Data Availability

The data presented in this study are available upon reasonable request from the corresponding author.

## Supplementary Materials

The following supporting information can be downloaded at: https://www.mdpi.com/article/10.3390/diagnostics16091376/s1, Table S1: Participant demographics and baseline characteristics; Table S2: Participant knowledge of AI in any healthcare setting, in breast clinics, and participant trust in AI findings; Table S3: Participant comfort with AI use in breast care; Table S4: Participant concerns and facilitators for AI use in healthcare.

## Abbreviations

The following abbreviations are used in this manuscript:

AI	Artificial Intelligence
Df	Degrees of Freedom
HRA	Health Research Authority
IBCs	Interval Breast Cancers
NSC	National Screening Committee
UK	United Kingdom
