# Supplementary material for: Patient and Public Perceptions of Artificial Intelligence in Breast Imaging and Clinical Decision-Making: An Exploratory Cross-Sectional Survey Study
Source: Diagnostics (Basel). 2026 May 1;16(9):1376. doi: 10.3390/diagnostics16091376 (PMC13163855; doi:10.3390/diagnostics16091376)
Supplement: Supplementary file 1 [file diagnostics-16-01376-s001.zip › diagnostics-4214865-supplementary.pdf]

Supplementary Materials

Table S1. Participant demographics and baseline characteristics.

Baseline characteristics, total n	n, %
**Patient or Public, 116**	
Patient	85 (73.3)
Family member	7 (6)
Carer	24 (20.7)
**Age (years), 117**	
18-24	4 (3.4)
25-35	14 (12)
36-45	21 (18)
46-60	44 (37.6)
61-74	27 (23.1)
75-84	6 (5.1)
85+	1 (0.9)
**Gender, 115**	
Female	103 (89.6)
Male	12 (10.4)
**Previous diagnosis, 119**	
Yes	33 (27.7)
No	66 (55.5)
No but have had concerns	20 (16.8)
**Comfort with technology, 120**	
Very comfortable	67 (55.8)
Fairly comfortable	31 (25.8)
Neither comfortable nor uncomfortable	7 (5.8)
Uncomfortable	11 (9.2)
Very uncomfortable	4 (3.3)
Don't know	0

Table S2. Participant knowledge of AI in any healthcare setting, in breast clinics, and participant trust in AI findings.

	n, %
**Knowledge of AI use in healthcare, n=120**	
Yes	74 (61.7)
No	40 (33.3)
Not sure	6 (5)
**Knowledge of AI use in breast clinics, n=120**	
I know a lot	4 (3.3)
I know a little	23 (19.2)
I don't know	54 (45)
I haven't heard about it	42 (35)
**Trust in AI findings, n=119**	
Trust completely	4 (3.4)
Trust a fair bit	40 (33.6)
Neutral	26 (21.9)
Trust a little	12 (10.1)

No trust at all	15 (12.6)
Don't know	22 (18.5)

Table S3. Participant comfort with AI use in breast care.

	n, %
**Comfort with AI use, alongside doctors, in interpreting mammograms and ultrasounds, n=120**	
Very comfortable	30 (25)
Fairly comfortable	30 (25)
Neither comfortable nor uncomfortable	27 (22.5)
Uncomfortable	14 (11.7)
Very uncomfortable	7 (5.8)
Don't know	13 (10.8)
**Comfort with AI use for deciding urgent from routine appointments, n=119**	
Very comfortable	23 (19.3)
Fairly comfortable	30 (25.2)
Neither comfortable nor uncomfortable	30 (25.2)
Uncomfortable	21 (17.7)
Very uncomfortable	5 (4.2)
Don't know	11 (9.2)
**Would you be happy for AI to be used in your breast care in the future, n=118**	
Yes, definitely	23 (19.5)
Yes, to some extent	59 (50)
No, not at all	9 (7.6)
Don't know	28 (23.7)

Table S4. Participant concerns and facilitators for AI use in healthcare.

	n, %
**What would help you feel more comfortable about AI use in your healthcare, n=118**	
More information about how AI works	47 (39.8)
Doctors checking and confirming AI findings	92 (78)
Knowing it has been tested in large settings	50 (42.4)
Don't know	9 (7.6)
I don't have any concerns	1 (0.9)
**What concerns would you have about AI being**	

**used in your healthcare, n=117**	
Mistakes/being misdiagnosed	86 (73.5)
Privacy of my details	46 (39.3)
Replacing doctors	74 (63.3)
I don’t have any concerns	6 (5.1)

Questionnaire

Artificial Intelligence (AI) in Breast Clinics
Patient Feedback

We are keen to hear your thoughts regarding Artificial Intelligence (AI) being included in our Breast Clinics.
Please complete the survey below. Your responses are anonymous and will be used to improve our services.

Date of visit: ______________________

Q1. Have you ever been diagnosed with breast cancer?
☐ Yes ☐ No ☐ No but have had concerns

Q2. How comfortable are you in using technology e.g. smartphones, computers etc?
☐ Very comfortable ☐Fairly comfortable ☐ Neither comfortable nor uncomfortable
☐ Uncomfortable ☐Very uncomfortable ☐ Don’t know

Q3. Before today, had you heard about AI (artificial intelligence) being used in any kind of healthcare setting?
☐ Yes ☐ No ☐ Not sure

Q4. Do you know how Artificial Intelligence (AI) may be used in Breast Clinics?
☐ I know a lot about how AI maybe used in Breast Clinics
☐ I know a little about how AI may be used in Breast Clinics
☐ I don't know anything about how AI may be used in Breast Clinics
☐ I haven't heard about AI being used in Breast Clinics

Q5. How comfortable would you feel if Artificial Intelligence (AI) was used alongside doctors to interpret your mammogram or ultrasound scan?
☐ Very comfortable ☐ Fairly comfortable ☐ Neither comfortable nor uncomfortable
☐ Uncomfortable ☐ Very uncomfortable ☐ Don't know

Q6. How comfortable would you feel if Artificial Intelligence (AI) helped to decide urgent from routine appointments?
☐ Very comfortable ☐ Fairly comfortable ☐ Neither comfortable nor uncomfortable
☐ Uncomfortable ☐ Very uncomfortable ☐ Don't know

Q7. How much would you trust Artificial Intelligence (AI) findings?
☐ Trust completely ☐ Trust a fair bit ☐ Neutral
☐ Trust a little ☐ No trust at all ☐ Don't know

Q8. What concern/s would you have, if any, about having Artificial Intelligence (AI) in your healthcare? (tick all that apply)
☐ Mistakes/being misdiagnosed ☐ Privacy of my details
☐ Replacing doctors ☐ I don't have any concerns
☐ Other, please specify:____

Q9. Is there anything that would help you feel more comfortable about Artificial Intelligence (AI) being used in your healthcare? (tick all that apply)

☐ More information on how AI works
☐ Doctors checking and confirming AI findings
☐ Knowing it has been tested in large settings
☐ Don't know
☐ I don't have any concerns
☐ Other, please specify: ____________________

Q10. Would you be happy for Artificial Intelligence (AI) to be used in your Breast Care in the future?

☐ Yes definitely ☐ Yes to some extent
☐ No not at all ☐ Don't know

Q11. We welcome any comments you would like to make regarding Artificial Intelligence (AI) being used in your Breast Clinic.

☐ Please tick this box if you do not wish your comments to be made public.

Tell us a little about you? (optional)
These questions ask about you. Your answers will help us understand whether experiences vary between different groups of the population.
Your answers will be kept confidential and not linked to your medical records.

Are you a
☐ Patient ☐ Carer ☐ Family member

What is your age?
☐ 16-24 ☐ 25-35 ☐ 36-45 ☐ 46-60 ☐ 61-74 ☐ 75-84 ☐ 85+

What is your gender?
☐ Female ☐ Male ☐ Transgender
☐ Prefer to self-describe ☐ Prefer not to say ☐ Non Binary

Thank you for completing this survey.
